# Metabolic reprogramming of three major nutrients in platinum-resistant ovarian cancer

**DOI:** 10.3389/fonc.2023.1231460

**Published:** 2023-08-22

**Authors:** Jinbowen Yan, Fangzhi Xu, Dan Zhou, Shuo Zhang, Bo Zhang, Qingwei Meng, Qiubo Lv

**Affiliations:** ^1^ Department of Obstetrics and Gynecology, Beijing Hospital, National Center of Gerontology; Institute of Geriatric Medicine, Chinese Academy of Medical Sciences, Beijing, China; ^2^ The Key Laboratory of Geriatrics, Beijing Institute of Geriatrics, Institute of Geriatric Medicine, Chinese Academy of Medical Sciences, Beijing Hospital, National Center of Gerontology of National Health Commission, Beijing, China

**Keywords:** ovarian cancer, platinum resistance, metabolic reprogramming, glucose metabolism, lipid metabolism, amino acid metabolism

## Abstract

Metabolic reprogramming is a phenomenon in which cancer cells alter their metabolic pathways to support their uncontrolled growth and survival. Platinum-based chemotherapy resistance is associated with changes in glucose metabolism, amino acid metabolism, fatty acid metabolism, and tricarboxylic acid cycle. These changes lead to the creation of metabolic intermediates that can provide precursors for the biosynthesis of cellular components and help maintain cellular energy homeostasis. This article reviews the research progress of the metabolic reprogramming mechanism of platinumbased chemotherapy resistance caused by three major nutrients in ovarian cancer.

## 1 Introduction

In gynecologic cancer, OC is ranked 2nd in mortality (1). Among ovarian malignancies, epithelial ovarian cancer (EOC) accounts for 90% (2). Since most patients are diagnosed at an advanced stage (3), the first-line primary treatment regimen recommended by the NCCN guidelines is surgery followed by platinum-based chemotherapy (4). Nevertheless, 25% of patients occurs platinum resistance at first recurrence (5). Patients who recrudesce within 6 months after the end of platinum-based first-line therapy are classified as being platinum-resistant, with a poor response to subsequent chemotherapy and median overall survival of 9-12 months (6). Therefore, the mechanism of platinum resistance and its influencing factors have been actively studied and explored, and new treatment options have been proposed to improve the prognosis of patients by finding key targets.

Cisplatin utilizes DNA as its most critical target of action to exert its cytotoxic effects by forming DNA adducts (7). The mechanisms of platinum-based chemotherapy resistance in ovarian cancer are complex and multifactorial, involving genetic and epigenetic changes, alterations in drug transport and metabolism, and activation of cellular survival pathways (8–15). Recent studies have shown that metabolic reprogramming plays a crucial role in the development of platinum-based chemotherapy resistance in ovarian cancer (16).

Metabolic reprogramming is a phenomenon in which cancer cells alter their metabolic pathways to support their uncontrolled growth and survival (17, 18). Platinum-based chemotherapy resistance is associated with changes in glucose metabolism, amino acid metabolism, fatty acid metabolism, and tricarboxylic acid cycle. These changes lead to the creation of metabolic intermediates that can provide precursors for the biosynthesis of cellular components and help maintain cellular energy homeostasis (19). This article reviews the research progress of the metabolic reprogramming mechanism of platinum-based chemotherapy resistance caused by three major nutrients in ovarian cancer.

## 2 Glucose metabolism

In the 2020s, Warburg’s findings revealed that even under aerobic conditions, tumor cells preferentially utilize glycolysis, known as aerobic glycolysis, instead of oxidative phosphorylation (20, 21). This metabolic reprogramming in tumor cells is not aimed at increasing energy production, but rather at generating raw materials for the synthesis of biological macromolecules, which support cell proliferation under limited resources. Recent studies have demonstrated that oncogenes and various tumor regulators can modulate key proteins and rate-limiting enzymes involved in glucose metabolism in tumor cells, promoting their growth and survival (22–24).

Key substances in the glucose metabolism process of ovarian cancer cells, including glucose transporter 1, hexokinase 1 (HK1), hexokinase II (HK2), etc., promote the proliferation and metastasis of tumor cells through a variety of downstream target genes and signaling pathways (25–32). In platinum-resistant ovarian cancer, the dysregulation of glucose metabolism intermediates, key enzymes and abnormally active metabolic activities play a significant role in promoting drug resistance in tumor cells.

### 2.1 Serine/threonine kinase Aurora-A

In a study, the researchers utilized the organoid model of ovarian cancer to validate the hypothesis that abnormally overexpressed serine/threonine kinase Aurora-A directly phosphorylates sex-determining region Y-box 8 (SOX8) at Ser327 or indirectly enhances SOX8 transcription via c-Myc (33). Subsequently, SOX8 targets HK2 and lactate dehydrogenase (LDHA), which affect the glycolysis process via the SOX8/FOXK1 axis, ultimately leading to drug resistance (**Table 1**)

**Table 1:** The alternation of molecules or pathways.

Molecular	Alternation	Pathway	Target alternation
Glucose metabolism			
Aurora-A	Overexpress	SOX8/FOXK1 axis	HK2 LDHA
Autophagy	Promoted by cisplatin-induced	ERK1/2	HK2
PGC1α	Overexpress	HSP70/HK2/VDAC1	HK2
PDK1	Overexpress	EGFR	
PXGDH	Decrease		Decreased serine biosynthesis Regenerative phenotype of NAD+
SIRT1	Increase		Promote OXPHOS
TRAP1 chaperone protein	Downregulation		Promote OXPHOS Stimulate inflammation response
Lipid metabolism			
NKX2-8	Genetic ablation		
DGKA	Activation	Recruit c-JUN	
FABP4	Overexpress		
Arachidonic acid	Adipocytes secrete		Inhibit cisplatin-induced apoptosis
FDPS OCS	Downregulation		LDLR overexpress
ARL4C	Overexpress		OSBPL5 overexpress
ABCG2 MDR1 LXRα	Overexpress		
Amino acid metabolism			
Glutamine synthetase	Regulated by DNA methylation		Preferentially GSH synthesis
GSH	Decrease	GSH-cisplatin adducts	Cysteine protective effect
SLC7A1/CAT1	Overexpress		Metabolic reprogramming of arginine
IDO1	Overexpress	ROS/p53	Arginine transport

### 2.2 Hexokinase II

**Figure 1:**
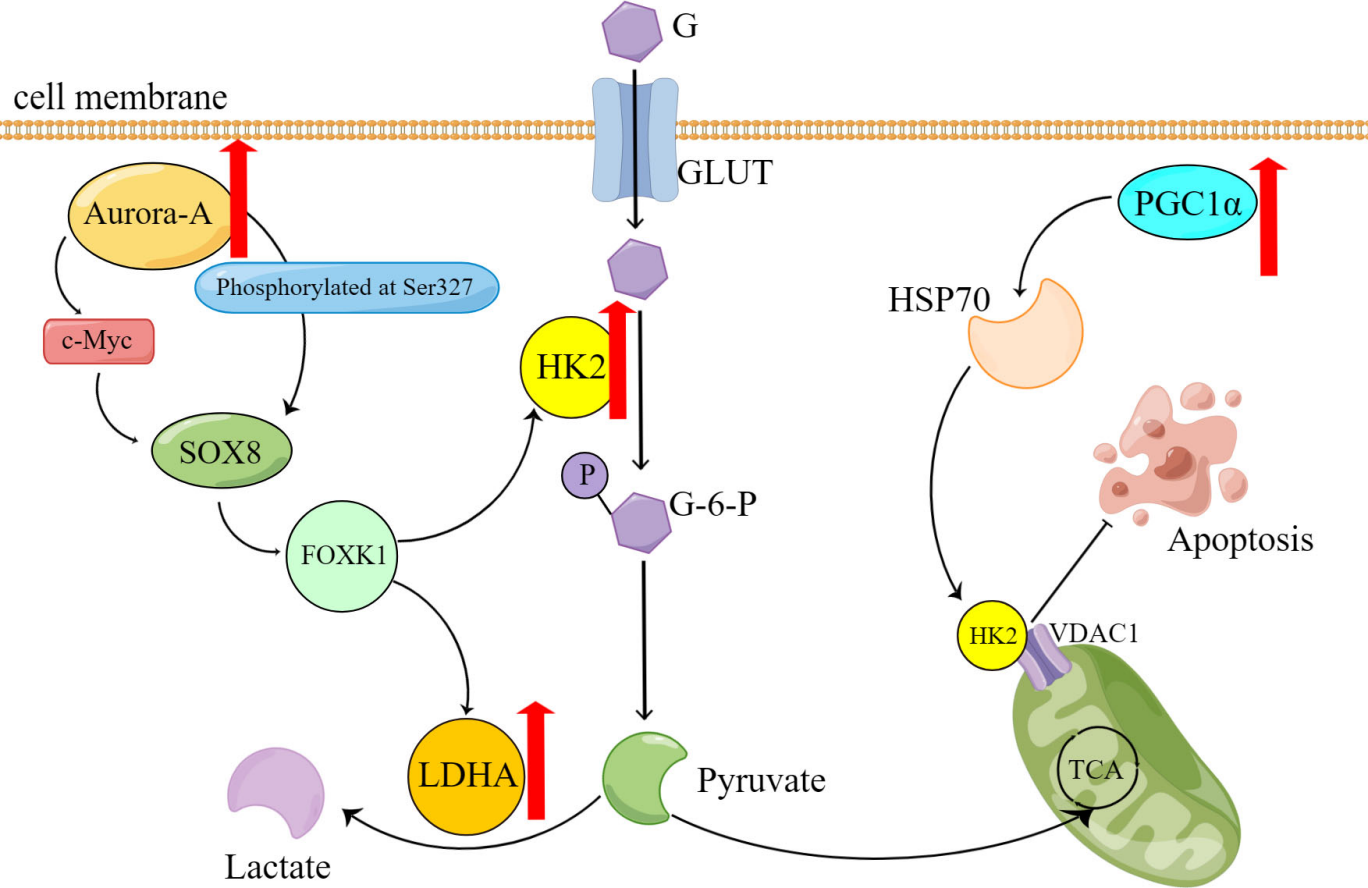
Overexpression of Aurora-A and PGC1α causes reprogramming of glucose metabolism and contributes to the onset of platinum resistance in ovarian cancer cells. G, glucose; GLUT, Glucose transporter; G-6-P, Glucose-6-phosphate; P, phosphate; TCA, tricarboxylic acid cycle.

Zhang XY et al. (34) shows that HK2 presents resistance to cisplatin in the ovarian cancer cell, promotes the resistance by enhancing cisplatin-induced autophagy, and boosts the cisplatin-induced autophagy by activating the ERK1/2 pathway. The upregulation of HK2 expression and subsequent drug resistance can be attributed to the effects of lysophosphatidic acid (LPA) (35). HK2 is expressed on mitochondria in cancer cells and interacts with voltage-dependent anion channel 1 (VDAC1) to impede apoptosis (**Figure 1**). Notably, studies have demonstrated that peroxisome proliferator-activated receptor coactivator 1 (PGC1α) (36), which is highly expressed in drug-resistant ovarian cancer cells, promotes the transcription of α heat shock protein (HSP70). Consequently, the upregulation of HSP70 leads to increased expression of HK2 on mitochondria, and its binding to VDAC1. Therefore, the HSP70/HK2/VDAC1 signaling pathway may serve as a potential target for therapies.

### 2.3 Pyruvate dehydrogenase kinase 1

Platinum-resistant ovarian cancer cells exhibit increased levels of pyruvate dehydrogenase kinase 1 (PDK1) in comparison to platinum-sensitive cells. The study by Zhang M et al. revealed that upregulated PDK1 activates epidermal growth factor receptor (EGFR) to facilitate the development of chemotherapy resistance (37). Notably, downregulating PDK1 in drug-resistant cells enhances the sensitivity to cisplatin-induced cell death. Despite these findings, the precise mechanism of action remains unclear.

### 2.4 Phosphoglycerate dehydrogenase

Van Nyen T, et al. (38) explored the relevance of phosphoglycerate dehydrogenase (PHGDH) expression and serine biosynthesis activity in tumors with acquired platinum resistance after platinum chemotherapy. They recognized a subset of ovarian cancer patients who relapsed after platinum-based chemotherapy with decreasing in PHGDH expression. Then, through *in vivo*, *in vitro*, and metabolic pathways tests, they investigated that this subgroup had undergone metabolic changes of decreased serine biosynthesis, which caused tumor cells to need exogenous serine for metabolic activities. This metabolic shift is accompanied by a regenerative phenotype of NAD+. Although tumor cells have no significant change in NAD+ levels, NAD+ regeneration is active and benefits from maintaining PARP’s activity to resistant platinum.

### 2.5 Mitochondrial oxidative phosphorylation

While the role of aerobic glycolysis in tumors has been extensively studied, recent research suggests that mitochondrial oxidative phosphorylation (OXPHOS) may also contribute to platinum resistance in ovarian cancer.

Platinum-based chemotherapy agents may induce an increase in NAD^+^-dependent deacetylase SIRT1 (39), which in turn promotes OXPHOS and enriches chemoresistant aldehyde dehydrogenase (ALDH) + ovarian cancer stem cells (OCSC), ultimately leading to drug resistance. However, the precise mechanisms underlying this phenomenon require further investigation. Furthermore, as a critical modulator of the mitochondrial respiratory chain (40), downregulation of the TRAP1 chaperone protein inhibits glycolysis in tumor cells and shifts metabolism towards OXPHOS, which in turn promotes the release of various inflammatory factors, including IL-6, to stimulate an inflammatory response that reduces platinum sensitivity in ovarian cancer cells (41).

### 2.6 Potential of metformin

As a classic treatment for diabetes, metformin may play a therapeutic role by exploiting the metabolic changes of platinum-resistant ovarian cancer, which is conducive to the cytotoxic effect of platinum drugs and prevents the progression of tumor cells. Metformin has been identified as a promising drug to overcome resistance to platinum-based therapy in ovarian cancer. The mechanism of action involves targeting mitochondrial complex I and ATP synthases, leading to a partial reversal of resistance (42). Metformin also regulates apoptosis by downregulating Bcl-2 and Bcl-xL expression and upregulating Bax and Cytochrome expression (43). Moreover, metformin has been shown to reduce inflammation and improve drug sensitivity by inhibiting the NFκB signaling pathway and IL-6 secretion (44, 45). Furthermore, metformin can reverse resistance via the p53-PDK1-HKII pathway and by activating the AKT signaling pathway while inhibiting the IGF2R signaling pathway (46, 47). Notably, metformin can also modulate the metabolic profile of resistant cells by upregulating taurine and histidine levels and downregulating tyrosine kinase levels, thus preventing chemoresistance (48, 49). In summary, the combination of metformin and cisplatin presents a promising therapeutic strategy for preventing drug resistance in ovarian cancer cells with metabolic alterations.

## 3 Lipid metabolism

Adipocytes and the tumor microenvironment formed with the participation of adipocytes have a very important impact on the occurrence, development, and prognosis of malignant tumors (50). Ovarian cancer is no exception (51). Aberrant expression of key enzymes, transport proteins, cellular receptors, and various adipocyte secretagogues (including cytokines, adipokines, and pro-inflammatory factors) in ovarian cancer promotes the formation of tumor cell microenvironment and increases the aggressiveness of ovarian cancer cells. For example, CD36 (52), one of the fatty acid translocases (FAT), contributes to the growth and distant metastasis of ovarian cancer cells; fatty acid binding protein 4(FABP4) contributes to the metastasis and implantation of ovarian cancer into the omentum (53). In turn, these key substances may serve as indicators for monitoring the process of platinum-based chemotherapy in ovarian cancer and as key targets for treatment after the development of platinum resistance.

### 3.1 β-oxidation

Fatty acid β-oxidation has been linked to platinum resistance (54), with genetic ablation of the homology box-containing developmental regulator NKX2-8 promoting fatty acid metabolism reprogramming and subsequent drug resistance in epithelial ovarian cancer cells in the adipose microenvironment (55). Additionally, a novel biomarker, Collagen type XI alpha 1 (COL11A1) (56), has been found to induce platinum resistance by upregulating fatty acid metabolism in ovarian cancer through its binding to discoid domain receptor 2 and activating Src-Akt-AMPK signaling. Furthermore, activation of the transcription factor c-JUN α diacylglycerol kinase (DGKA) leads to the recruitment of c-JUN N-terminal kinase to c-JUN and enhances the expression of the cell cycle regulator WEE1 under cisplatin stimulation, ultimately leading to platinum resistance in ovarian cancer (57).

### 3.2 Fatty acid binding protein 4

In the context of ovarian cancer, FABP4 plays a central role in regulating adipocyte-induced lipid metabolism in cancer cells (58). Its overexpression promotes tumor proliferation and metastasis and mediates drug resistance to carboplatin. However, the downstream factors and pathways that specifically mediate drug resistance of FABP4 remain unclear. Adipocytes can also inhibit cisplatin-induced apoptosis by secreting arachidonic acid, which activates Akt (59). Moreover, the SP1-12LOX axis can upregulate the expression of multidrug resistance-associated protein (MRP) via signal transduction, increase the production of arachidonic acid-derived metabolites, and promote drug resistance (60).

### 3.3 Cholesterol

Cholesterol metabolism dysregulation can be a contributing factor to platinum resistance in ovarian cancer, as cholesterol is a vital component of cell membranes and plasma lipoproteins and a precursor for important bioactive molecules (61). The decrease in TRAP1 expression, as mentioned earlier, also impacts cholesterol metabolism. In drug-resistant ovarian cancer cells, exogenous uptake becomes the primary source of cholesterol, with decreased expression of farnesyl bisphosphate synthase (FDPS) and oxidized squalene cyclase (OCS) involved in endogenous cholesterol synthesis, and increased expression of low-density lipoprotein receptor (LDLR) promoting exogenous cholesterol uptake (62). Furthermore, carboplatin-resistant ovarian cancer cells exhibit upregulation of ADP ribosylation factor 4C (ARL4C), a member of the ADP ribosylation factor subfamily, and Notch-RBP-Jκ-H5K3Me4, a member of the oxysterol-binding protein family (OSBP), resulting in the upregulation of OSBPL5, a member of the OSBP family, promoting cholesterol accumulation and autophagy, leading to carboplatin resistance (63). Malignant ascites exhibited elevated cholesterol levels, with upregulated drug efflux proteins ABCG2 and MDR1, and cholesterol receptor LXRα expression, contributing to multidrug resistance (64).

## 4 Amino acid metabolism

Assays of serum-free amino acids have shown that the levels of many amino acids change significantly during the development of ovarian cancer and that this change in turn promotes tumor development (65, 66). Amino acids that changed significantly compared to healthy women included histidine, alanine, asparagine, citrulline, cystine, ethanolamine, lysine, methionine, ornithine, threonine, and tryptophan (67). There are many mechanisms of tumorigenesis caused by changes in amino acid metabolism, such as regulating the invasion of ovarian cancer cells through transcription factor ETS1 in the case of glutamine deprivation (68); Six enzymes of folic acid metabolism pathway were overexpressed, among which serine hydroxymethyltransferase 1 promoted the expression of cancer inflammatory factors by regulating sialic acid N-acetylneuraminic acid, and promoted the growth and metastasis of ovarian cancer tumor cells (69); Upregulation of non-tyrosine kinase (FER) in ovarian cancer promotes tumorigenesis by activating the PI3K-AKT pathway through the kinase-substrate mode of action between FER and insulin receptor substrate 4 (IRS4) (70). Changes in amino acid metabolism may also contribute to the development of platinum resistance in ovarian cancer.

### 4.1 Glutamine

Glutamine, the most abundant non-essential amino acid in the human body, serves a multitude of purposes, including its function as a key substrate or molecule in the biosynthesis of various compounds, and its essential role in maintaining normal cellular function and integrity (71). Aside from relying on aerobic glycolysis, drug-resistant ovarian cancer cells exhibit an addiction to glutamine (72). DNA methylation has been found to regulate the expression of the critical enzyme glutamine synthetase, resulting in the metabolic reprogramming of glutamine in tumor cells (73). Preferentially directing glutamine towards glutathione (GSH) synthesis instead of the TCA cycle ultimately leads to increased levels of glutamine, glutamic acid, and glutathione. Despite the sensitivity of drug-resistant tumor cells to nutrient deficiencies, glutamine can combat starvation-induced cell death by increasing nucleotide concentrations (74).

### 4.2 Glutathione

**Figure 2:**
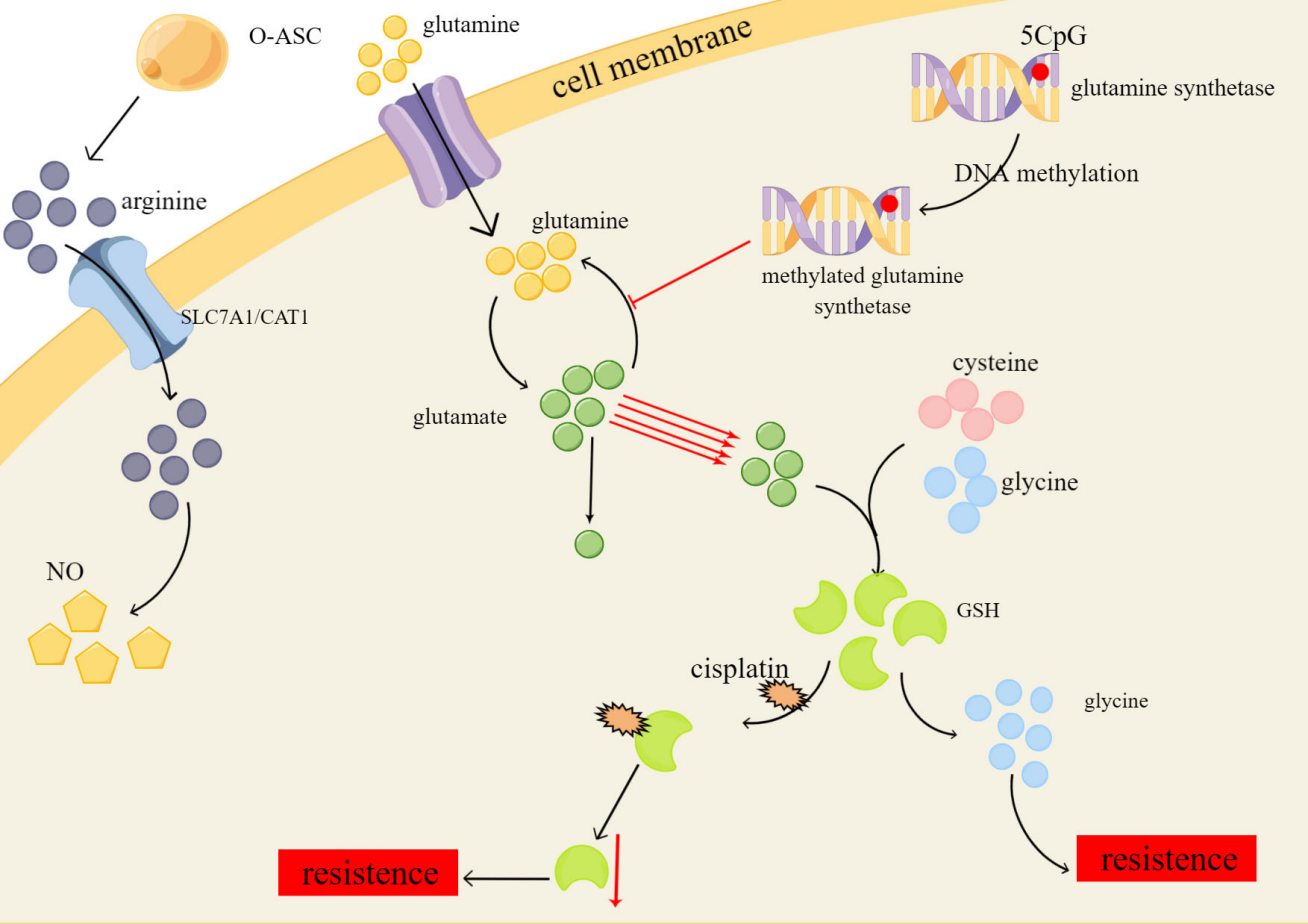
Platinum resistance caused by changes of glutamine, GSH and ariginine in amino acid metabolism of ovarian cancer. O-ASC, omental adipose stromal cells; SLC7A1/CAT1, cationic amino acid transporters; NO, nitric oxide.

GSH is synthesized from glutamic acid, cysteine, and glycine and is one of the important members of cellular antioxidants (75). GSH depletion in tumor cells can increase the attack of chemotherapy drugs on tumor cells through reactive oxygen species (ROS) accumulation, detoxification reduction, and iron death to achieve therapeutic purposes (76). Non-targeted metabolomics analyses showed that platinum-resistant ovarian cancer cells have significantly reduced levels of GSH, which contributes to chemoresistance by forming adducts with cisplatin, leading to reduced intracellular concentrations of the active drug, and cisplatin-GSH adducts also reduce intracellular GSH levels. And these resistant cells can activate alternative antioxidant defenses independent of GSH (77). Cysteine stored in large quantities in GSH has a protective effect on ovarian cancer cells in a hypoxic environment and can also make tumor cells resistant to carboplatin (78, 79) (**Figure 2**).

### 4.3 Other amino acids

In the context of arginine deficiency, Omental adipose stromal cells (O-ASC) have been shown to stimulate tumor cell growth and NO synthesis by secreting arginine (80). The uptake and transport of arginine across cell membranes are mediated by cationic amino acid transporters, specifically SLC7A1/CAT1, which are highly expressed in ovarian cancer and are involved in the metabolic reprogramming of arginine, ultimately promoting platinum resistance (81). Furthermore, studies have shown that ovarian cancer cells with elevated levels of indoleamine 2,3-dioxygenase 1 (IDO1), an enzyme that catalyzes the conversion of tryptophan to l-kynurenine (Kyn), exhibit platinum resistance by downregulating the ROS/p53 pathway (82). Hence, it is important to further investigate the role of arginine transport and IDO1 in the development of platinum resistance in ovarian cancer cells.

## 5 Summary

The metabolic processes of the three major nutrients are interrelated and influence each other. For example, glucose can be converted into fat, and the intermediate products of sugar metabolism can generate non-essential amino acids. Glycerol can be converted into sugar in the body, and most amino acids can be converted into glucose. Proteins can be converted into fats, amino acids can be used as raw material for phospholipid synthesis, and glycerol can be converted into non-essential amino acids. The metabolism between the three is linked by the tricarboxylic acid cycle. The changes in key enzymes, intermediates, upstream genes, and downstream targets in the process of being stimulated by cytotoxic drugs can cause changes in a variety of metabolic processes, resulting in changes in ovarian cancer cells and their tumor microenvironment. These changes can help tumor cells cope with the need for nutrients during growth. In addition to the three major nutrients, the metabolic changes of vitamins, metals, and trace elements may also lead to platinum resistance in tumors. At present, the clinical treatment of patients with platinum-resistant recurrent ovarian cancer is still a difficult problem, and the key substances in the process of growth and metabolism and their molecular pathways may be used as therapeutic targets to improve the prognosis and survival time of patients. In general, metabolic reprogramming is of great significance in finding the key points for the treatment of platinum-resistant relapse.

## Author contributions

All authors contributed to the article and approved the submitted version.
